# Supplementary material for: An Innovative Chiral UPLC-MS/MS Method for Enantioselective Determination and Dissipation in Soil of Fenpropidin Enantiomers
Source: Molecules. 2022 Oct 2;27(19):6530. doi: 10.3390/molecules27196530 (PMC9572594; doi:10.3390/molecules27196530)
Supplement: Supplementary file 1 [file molecules-27-06530-s001.zip › molecules-1944083-supplementary-.pdf]

Supplementary Material

An Innovative Chiral UPLC-MS/MS Method for Enantioselective Determination and Dissipation in Soil of Fenpropidin Enantiomers

Rui Li, Yanqing Zhang, Yanhong Li, Zihao Chen, Zhen Wang and Minghua Wang*

Department of Pesticide Science, College of Plant Protection, Nanjing Agricultural University, State & Local Joint Engineering Research Center of Green Pesticide Invention and Application, Nanjing 210095, China.

*Corresponding author: Email: wangmha@njau.edu.cn; Tel: +86 25 84395479; Fax: +86 25 84395479

Table S1. Effect of different proportion of buffer solution on the enantioselective separation of fenpropidin

buffer solution	Rs	response value (Peak 1)	response value (Peak 2)
0.05% ammonia solution	1.80	6116052	6004277
0.1% ammonia solution	1.96	6321188	6460030
0.2% ammonia solution	2.27	5727396	6068982

Table S2. Van't Hoff equations and thermodynamic parameters

Enantiomer	ln *k*	ln *α*	ΔΔH^0 kJ/mol	ΔΔS^0 J/(mol·k)
R	$\ln k_1 = 1363.1/T - 3.3741$ ($R^2 = 0.9941$)	$\ln \alpha = 356.75/T - 0.7676$ ($R^2 = 0.9966$)	-2.97	-6.38
S	$\ln k_2 = 1719.8/T - 4.1417$ ($R^2 = 0.9953$)			

Table S3. Influence of temperature on the enantioseparation of fenpropidin on Lux cellulose-3

Temperature/°C	t_1	k_1	t_2	k_2	α	Rs
20	12.97	3.55	18.71	5.56	1.57	2.17
25	12.43	3.36	17.59	5.17	1.54	2.17
30	11.64	3.08	16.07	4.64	1.50	2.16
35	11.03	2.87	14.97	4.25	1.48	2.09
40	10.40	2.65	13.79	3.84	1.45	2.00

Table S4. ANOVA of retention time (Y_1) and independent variables (X_1, X_2, and X_3)

Source	Sum of Squares	df	Mean Square	F-value	p-value	
Model	6.76	4	1.69	76.96	< 0.0001	significant
X_1	0.0764	1	0.0764	3.48	0.0866	
X_2	6.65	1	6.65	302.87	< 0.0001	
X_3	0.0000	1	0.0000	0.0008	0.9776	
X_3^2	0.0327	1	0.0327	1.49	0.2455	
Residual	0.2633	12	0.0219			
Lack of Fit	0.2602	8	0.0325	41.20	0.0014	significant
Pure Error	0.0032	4	0.0008			
Cor Total	7.02	16				

Tabe S5. ANOVA of resolution (Y_2) and independent variables (X_1, X_2, and X_3)

Source	Sum of Squares	df	Mean Square	F-value	p-value	
Model	1347.50	6	224.58	10.24	< 0.0001	significant
X_1	58.81	1	58.81	2.68	0.1325	
X_2	1272.10	1	1272.10	58.02	< 0.0001	
X_3	13.49	1	13.49	0.6154	0.4509	
X_1X_3	0.0529	1	0.0529	0.0024	0.9618	
X_1^2	2.51	1	2.51	0.1146	0.7420	
X_3^2	0.4096	1	0.4096	0.0187	0.8940	
Residual	219.27	10	21.93			
Lack of Fit	219.24	6	36.54	4334.42	< 0.0001	significant
Pure Error	0.0337	4	0.0084			
Cor Total	1566.76	16				

Table S6. Free binding energy of fenpropidin enantiomers to CSPs

CSPs	compoums	Glide Score (kcal/mol)
Lux cellulose-1	*R*	-4.476
	S	-4.470
Lux cellulose-2	*R*	-4.719
	S	-4.659
Lux cellulose-3	*R*	-5.092
	S	-4.755

Table S7. The half-lives of fenpropidin enantiomers in soil

	Compounds	Kinetic Equation	R^2	Half-life (d)	*p*
Soil	*S*-(-)-fenpropidin	$y = 0.7882e^{-0.031x}$	0.8728	22.4	0.001
	R-(+)-fenpropidin	$y = 0.8624e^{-0.035x}$	0.9121	19.8	

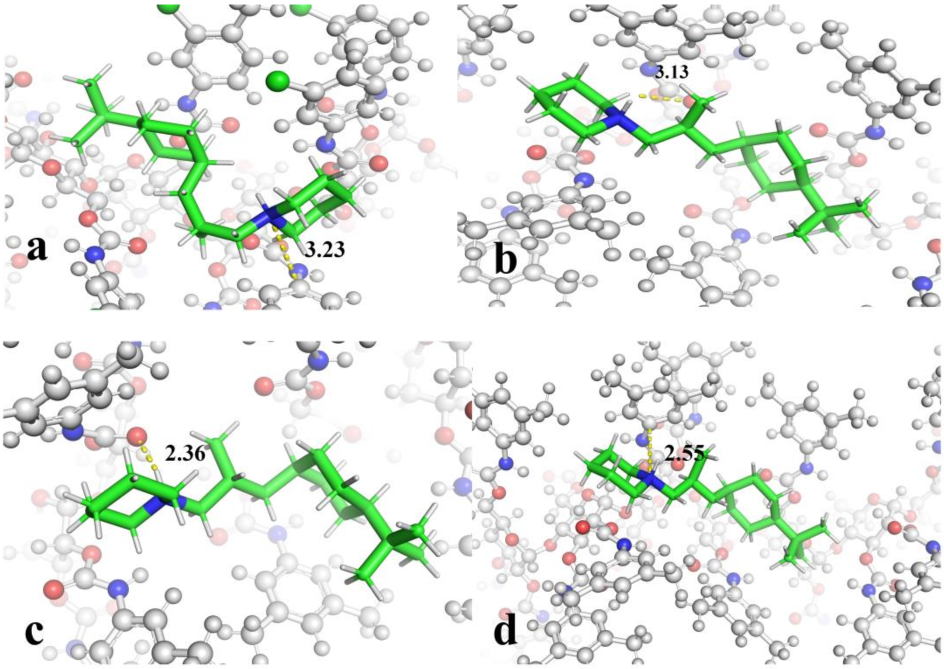

Figure S1. The docking posture of *R*-fenpropidin (a, c) and the posture of *S*-fenpropidin (b, d) with L1, L2.

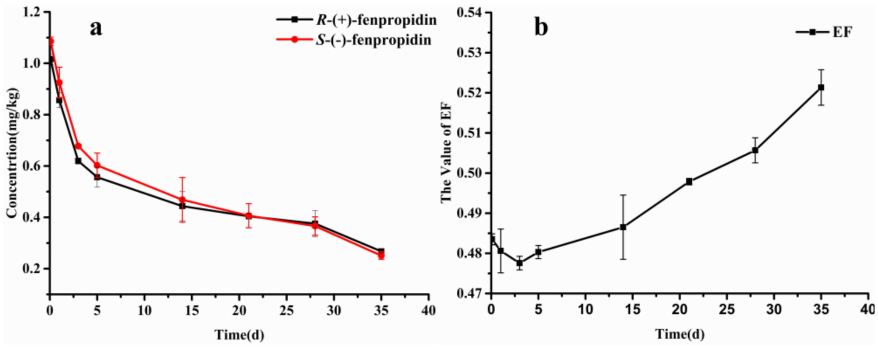

Figure S2. The dissipation trends (a) and EFs (b) of the fenpropidin in soil.
